# Characterization of human placenta-derived exosome (pExo) as a potential osteoarthritis disease modifying therapeutic

**DOI:** 10.1186/s13075-023-03219-z

**Published:** 2023-11-28

**Authors:** Chenfei Huang, Yuechao Zhao, Shengchen Lin, Lin Li, Xuan Guo, Sebastian Yumiseba, Jeng-dar Yang, Robert Hariri, Qian Ye, Shuyang He, Adrian Kilcoyne

**Affiliations:** Celularity Inc., 170 Park Avenue, Florham Park, NJ 07932 USA

**Keywords:** Osteoarthritis, Placenta, Exosome, Therapeutic

## Abstract

**Objective:**

Human placenta-derived exosomes (pExo) were generated, characterized, and evaluated as a therapeutic candidate for the treatment of osteoarthritis (OA).

**Methods:**

pExo was generated from full-term human placenta tissues by sequential centrifugation, purification, and sterile filtration. Upon analysis of particle size, cytokine composition, and exosome marker expression, pExo was further tested in cell-based assays to examine its effects on human chondrocytes. In vivo therapeutic efficacies were evaluated in a medial meniscal tear/medial collateral ligament tear (MCLT + MMT) rat model, in which animals received pExo injections intraarticularly and weight bearing tests during in-life stage while histopathology and immunohistochemistry were performed as terminal endpoints.

**Results:**

pExo displayed typical particle size, expressed maker proteins of exosome, and contained proteins with pro-proliferative, pro-anabolic, anti-catabolic, or anti-inflammatory activities. In vitro, pExo promoted chondrocyte migration and proliferation dose-dependently, which may involve its activation of cell growth-related signaling pathways. Expression of inflammatory and catabolic genes induced in a cellular OA model was significantly suppressed by pExo. In the rat OA model, pExo alleviated pain burden, restored cartilage degeneration, and downregulated expressions of pro-inflammatory, catabolic, or apoptotic proteins in a dose-dependent manner.

**Conclusions:**

Our study demonstrates that pExo has multiple potential therapeutic effects including symptom control and disease modifying characteristics. This may make it an attractive candidate for further development as an anti-OA therapeutic.

**Supplementary Information:**

The online version contains supplementary material available at 10.1186/s13075-023-03219-z.

## Introduction

Affecting over 300 million people worldwide as a degenerative disease, osteoarthritis (OA) is pathologically defined by articular cartilage destruction with whole-joint inflammation and damage. OA causes chronic joint pain and motion dysfunction symptomatically, which seriously reduce life quality and increase social burden [1]. In addition to surgical and non-pharmacological managements, pharmacological treatments for OA are aimed at achieving pain control to improve function and quality of daily life [2]. However, among the few approved pharmacological treatments such as intraarticular (IA) corticosteroids so far, no such therapies have been proven to modify the disease pathology by inhibiting the catabolic pathways, suppressing inflammation, or promoting regeneration [3], highlighting the largely unmet clinical needs of treating OA.

The current understanding of OA as a complex and multifactorial disease process facilitates the development of novel therapies, especially those with modulation of multiple targets. For example, cell-based approaches using autologous chondrocytes [4], induced pluripotent stem cells [5], or mesenchymal stromal/stem cells (MSCs) [6] demonstrated promising results both preclinically and in OA patients. Furthermore, various types of MSC-derived exosomes, which are defined as secreted bi-lipid membranes and nano-sized vesicles, have shown multiple effects including immunosuppression and tissue repair [7]. By mediating MSCs’ biological effects, exosomes may serve as a novel approach for OA treatment based on recent primary research [8] and clinical practice [9]. Exosomes can also overcome clinical limitations of cellular therapies such as tumorigenicity, immunogenicity [10], and operational challenges of cell product handling and storage [11]. Therefore, MSC exosomes are gradually becoming a promising strategy for OA.

The placenta is a highly specialized and essential organ for mammalian reproduction and a unique source of MSCs [12–14]. Human placental-derived biomaterials have been increasingly recognized for their therapeutic applications across multiple diseases with consistent safety and efficacy profiles being observed [15]. For example, Interfyl®, a decellularized flowable placental connective tissue matrix product, is applied in wound healing [16], while BIOVANCE®, a decellularized and dehydrated human amniotic membrane allograft, supports tissue repair [17]. Serial studies have investigated different human placental tissue products in animal models of OA [18–23]. Furthermore, exosomes secreted by cultured amniotic fluid stem cells (AFSCs) were reported to support a regenerative and less pro-inflammatory phenotype in a chemical-induced OA model [24]. Nevertheless, the therapeutic effects of exosomes directly isolated from placentas have not been studied in OA so far.

Considering the above evidence and modulation of maternal–fetal tolerance during pregnancy by placenta-derived exosomes (pExo) [25], we hypothesize that pExo may help repair OA-associated cartilage damage and suppress inflammation. Therefore, pExo was generated, characterized, and evaluated for its therapeutic effects in vitro and in vivo. Our results highlight the potential of pExo as a disease-modifying therapeutic against OA.

## Methods

### Preparation of pExo

**Fig. 1:**
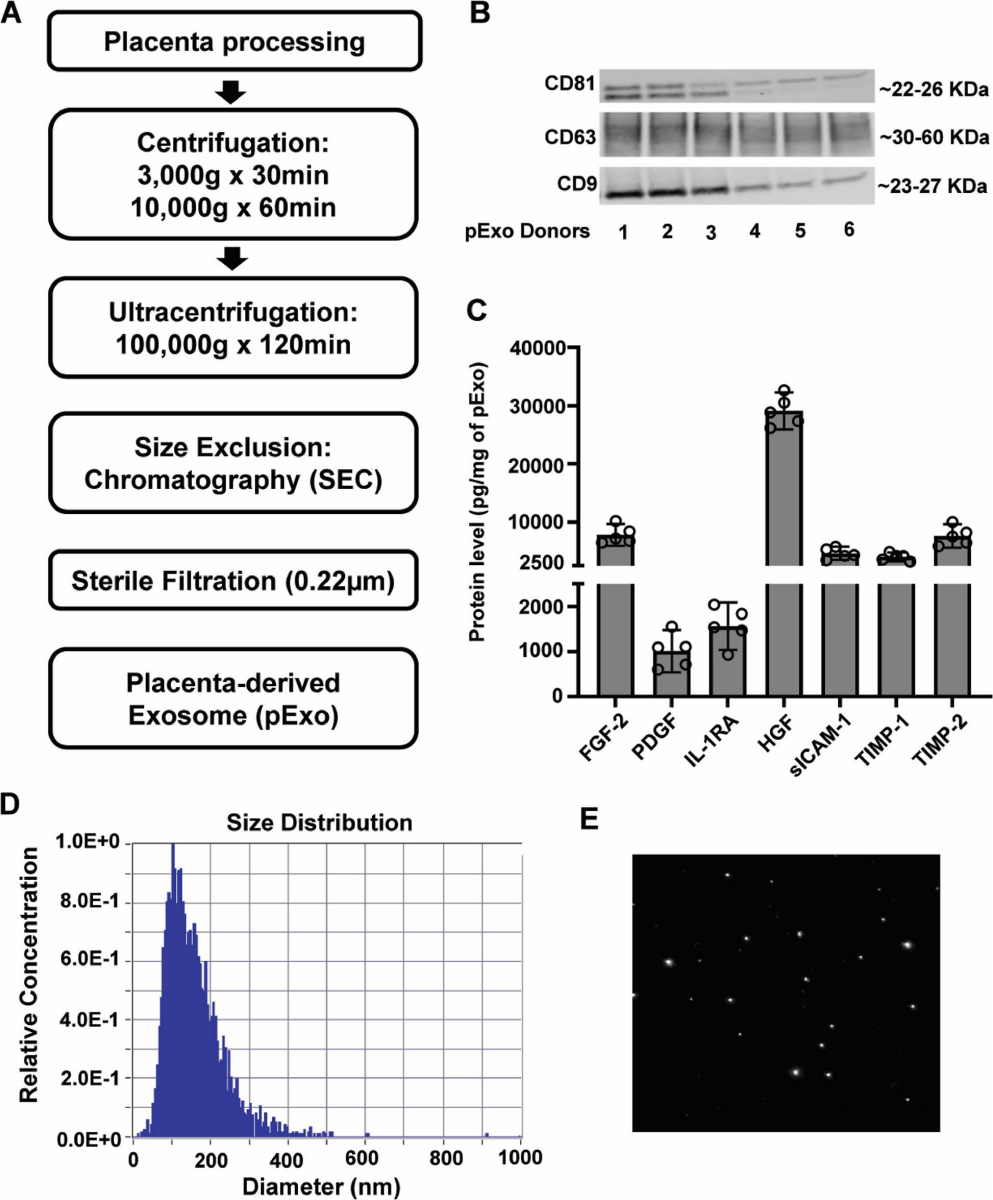
Generation and characterization of pExo. **A** Flow chart of pExo generation. **B** Western blots of pExo (*N* = 6 donors) to examine the levels of CD81, CD63, and CD9 as exosome markers. **C** Protein composition analysis showed the levels of OA-relevant proteins in pExo (*N* = 5 donors). Data is presented as mean ± 95% CI. Size distribution diagram (**D**) and particle image (**E**) were generated by NTA from a representative pExo donor

As summarized in Fig. 1A, full-term healthy human placentas procured through donors with full consent were dissected and incubated with saline in sterile cell culture containers at room temperature (RT) for 2 h. The supernatant underwent sequential centrifugations at 3000, 10,000, and 100,000* g*. The centrifuged pellets were defined as crude pExo and purified by size exclusion chromatography (SEC) using qEV10/35 nm Legacy Column Gen 1 (Izon, Product Code SP7). Purified pExo fractions were sterilized through 0.22-μm filters and diluted in saline (vehicle), from which the concentrations were measured by Micro BCA protein assay kit (Thermo Fisher Scientific, Cat# 23,235). The size distribution of pExo was determined by nanoparticle tracking analysis (NTA) using a ZetaView particle tracker (Particle Metrix GmbH, Germany).

### Western blot

Protein samples of 2 μg pExo were loaded, separated on 4–12% SDS gels (Novex Wedgewell, Invitrogen, Cat# NP0321BOX), and followed by blocking for 30 min in 3% blocker BSA solution and overnight incubation with primary antibodies (Thermo Fisher Scientific) of CD9 (Cat# 10626D), CD63 (Cat# 10628D), and CD81 (Cat# 10630D) at 4 °C. The membranes were then washed using Tris-buffered saline with 0.1% Tween on a plate shaker and incubated with IRDye 680RD secondary antibody (LiCOR Biosciences, Cat# 926–68,071) for 1 h at RT. Odyssey CLX (LI-COR Biosciences) was used to visualize blotting in the membranes.

### Protein composition analysis

Protein samples of 1–2 μg pExo determined by Micro BCA protein assay kit (Thermo Fisher Scientific, Cat# 23,235) were lysed according to the manufacturer’s instruction. pExo were placed in 50 μl RIPA buffer, incubated at 4 °C for 30 min, placed in an ice-cold sonication bath for 30 s, and followed by a gentle mix on ice for 15 min. cOmplete™ EDTA-free Protease Inhibitor (Sigma Aldrich, Schnelldorf, Germany) was used during the lysis. The same volume of pExo lyses (*N* = 5 donors) were loaded to analyze the protein composition by multiplex assays (MILLIPLEX® Human Cytokine/Chemokine Magnetic Bead Panel, Millipore Sigma, Cat# HCYTMAG-60 K-PX41). TIMP-1 and TIMP-2 levels were assayed using Human TIMP Magnetic Bead Panel 2 (Millipore Sigma, cat# HTMP2MAG-54 K). Following the vendor’s instruction, the median fluorescence intensity (MFI) of all beads was measured for quantification.

### Human chondrocyte culture and WST-1 assay

Primary articular chondrocytes were purchased from Lonza (Cat# CC-2550) and expanded in the vendor’s culture media (Cat# CC-3216). To evaluate proliferation, chondrocytes were starved in basal media (BM) for 24 h and treated with 50 or 200 μg/ml pExo for 72 h in 96-well plates (1 × 10^4^ cells/well). By the end, the WST-1 reagent (Roche, cat# 11,644,807,001) was added for 4 h before optical density (OD) absorbance measurement at 440 nm. Assays were performed in triplicate.

### Transwell cell migration assay

Chondrocytes were seeded on the top chamber of an 8-μm Transwell (Corning, cat# 3422) on a 24-well plate with 500 μl BM with or without pExo in the bottom chamber. After 12 h, non-migrated cells on the top chamber were removed with a cotton swab, and the Transwells were stained with crystal violet. Migrated cells passed through the membrane were counted under a microscope. Five representative areas were counted per Transwell. Five areas of each Transwell were counted, and assays were performed in triplicate.

### Signaling pathway analysis

After 24-h starvation in serum-free media, chondrocytes in 6-well plates (1 × 10^6^ cells/well) were treated with pExo (100 μg/ml) or growth factors (100 ng/ml) for 30 min. To analyze the signaling pathways using MILLIPLEX Multi-Pathway Magnetic Bead 9-Plex (Millipore Sigma, cat# 48-680MAG), chondrocyte lysates were prepared following the manufacturer’s instruction, and MFI of all beads was measured for quantification of target protein phosphorylation levels. Assays were performed in triplicate.

### Quantitative real-time PCR (qRT-PCR) assay

Total RNAs of chondrocytes treated with pExo (100 μg/ml) were isolated and used for gene expression analysis using Taqman probes purchased from Thermo Fisher Scientific: MMP13 (Hs00942584_m1), NOS2 (Hs01075529_m1), IL6 (Hs00174131_m1), TNFA (Hs00174128_m1), and GAPDH (Hs02786624_g1). Assays were performed in triplicate. The fold change of each gene’s expression was internalized with that of GAPDH.

### Rat OA study

In vivo effects of pExo were evaluated in the medial collateral ligament transection and medial meniscus tear (MCLT + MMT) surgery-induced OA model, which is relevant to test chondroprotective interventions [26], using healthy male Sprague–Dawley rats (10–12 weeks old), as approved by Institutional Animal Care and Use Committee (IACUC) of Pharmaseed (Ness-Ziona, Israel) with controlled order of treatments and measurements to minimize potential confounders. Briefly, rats were housed in polyethylene cages (three rats/cage), included, randomized, and stratified into four groups according to the static weight bearing test (Additional file 1: Supplemental Methods) results on day 0. The MCLT + MMT surgery was performed on day 1: an incision on the medial side of the patellar tendon provided access to the joint that was exposed; the medial ligament was transected, and the medial meniscus was resected under a surgical microscope. In sham-operated rats (*N* = 6 rats), the right knee joint was exposed and then closed without medial ligament transection or meniscus resection.

Vehicle (saline, 30 µl/injection, *N* = 11) or pExo (20 µg/injection) was given by either single (*N* = 10) or triple (*N* = 10) IA injections to the operated leg on days 5, 12, and 19 as illustrated in Fig. 5A. The number of groups and the total number of animals were based on previous studies demonstrating that this is the minimum number of animals per group required to obtain indicative information. Clinical observations (Additional file 1: Supplemental Methods), body weight, and pain measurements based on static weight bearing tests were performed weekly until termination on day 44. Knee joint samples were collected, fixed, and processed for toluidine blue (TB) staining-based histopathology (Additional file 1: Supplemental Methods) and immunohistochemistry (IHC) evaluation. The conduct of experiments and measurements at the in-life stage and terminal analyses were performed in a blind manner without technicians being aware of group allocation.

### IHC analysis

Three paraffin sections were cut from each knee, dewaxed, dehydrated, and treated with 3% H_2_O_2_ (Thermo Fisher Scientific, Cat# 426,001,000) to quench endogenous peroxidase activity. Antigen retrieval was performed using Histo/Zyme solution (Sigma, Cat# H3292-15ML). The sections were incubated with primary antibodies (Additional file 1: Table S2) overnight at 4 °C then horseradish peroxidase-conjugated secondary antibodies (Vector, Cat# PK-6101 & PK-6102) for 1 h at RT. Hydrogen peroxide, NovaRed (Vector, Cat# SK-4805), and hematoxylin (Vector, cat# H-3401) were used as substrate, dye, and counterstaining, respectively. Images were taken with ZEISS Axiocam microscope cameras. The staining intensity of MMP8 and COL-II was quantified by using the ImageJ software. For CCP3, iNOS, TNFα, and IL6 quantification, positively stained and non-stained cells were counted at × 100 magnification in three fields of each section and presented as percentages of positive cell number of total cell number.

### Statistical analysis

GraphPad Prism 9.3.1 (GraphPad Prism Software, Inc.) was used to calculate the statistics generated by *T*-test and one- or two-way ANOVA with Tukey’s multiple comparisons test.

## Results

### pExo possesses exosome characteristics and contains OA-relevant proteins

After preparation (Fig. 1A), pExo was confirmed to carry exosome-associated signature proteins CD9, CD63, and CD81 [27] by Western blots (Fig. 1B and Additional file 1: Fig. S1). Based on the NTA results (Additional file 1: Table S1), pExo derived from multiple donors exhibited an average range of 177 ± 49 nm in size, consistent with the previously described exosome size ranging from 50 to 200 nm [28]. Furthermore, the protein composition analysis (Fig. 1C) revealed pExo contained significant levels of molecules related to OA therapeutic or healing: fibroblast growth factor 2 (FGF-2), platelet-derived growth factor (PDGF), and hepatocyte growth factor (HGF) to promote chondrocyte proliferation and proteoglycan synthesis [29–31], soluble intercellular adhesion molecule-1 (sICAM-1) as an inflammation regulator [32], tissue inhibitor of metalloproteases (TIMP)-1 and 2 as anti-catabolic factors [33], and interleukin-1 receptor antagonist (IL-1RA) which has been investigated as an OA therapeutic target [34].

### pExo promotes chondrocyte proliferation and migration

**Fig. 2:**
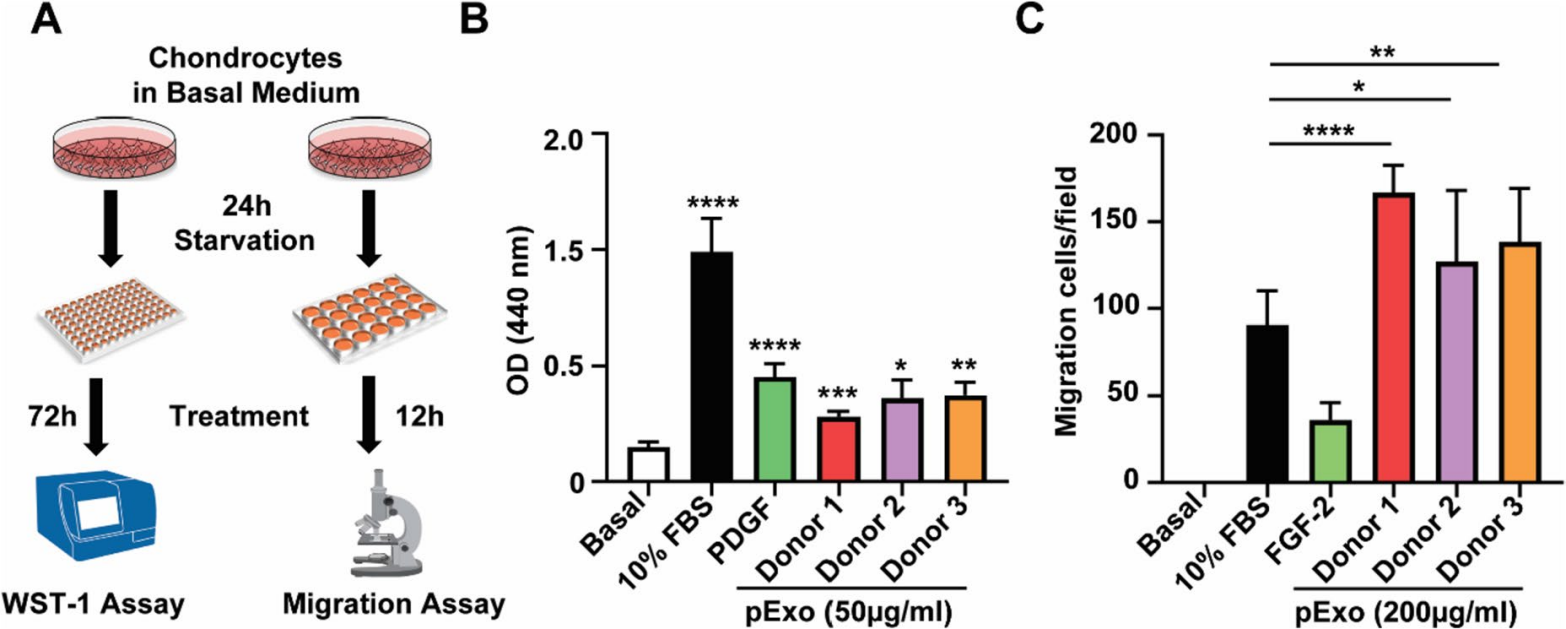
Effects of pExo in chondrocytes. **A** In vitro study scheme to determine the impacts of pExo on chondrocytes. **B** WST-1 assay to compare the impacts of 10% FBS, PDGF (100 ng/ml), or pExo (*N* = 3 donors, 50 µg/ml) on chondrocyte proliferation. Data is presented as mean ± 95% CI (*t*-test, vs basal medium condition, *P < 0.05, **P < 0.01, ***P < 0.005, ****P < 0.0001). **C** Traswell migration assay to evaluate the effects of 10% FBS, FGF-2 (100 ng/ml), or pExo (*N* = 3 donors, 200 µg/ml) for chondrocyte migration. Data is presented as mean ± 95% CI (*t*-test, vs 10% FBS condition, *P < 0.05, **P < 0.01, ****P < 0.0001)

MSC-derived exosomes are reported to promote chondrocyte proliferation, one of the hallmarks of OA prevention and protection [35]. In our study, human chondrocytes were actively proliferating in the presence of nutrient factor-enriched 10% FBS, while pExo and PDGF at indicated concentrations showed comparable abilities in promoting chondrocyte proliferation (Fig. 2A, B). Of note, pExo exhibited this pro-proliferative effect in chondrocytes in a dose-dependent manner within 1–200 µg/ml (Additional file 1: Fig. S2). Chondrocyte’s migration ability is also known to be essential for cartilage repair [36]. We found that chondrocytes did not migrate in BM condition at the time point monitored (Additional file 1: Fig. S3), while these cells showed even more migration when treated with pExo compared to 10% FBS (Fig. 2C). Collectively, these in vitro results demonstrate the promoting effects of pExo in chondrocytes.

### pExo activates cell growth signaling pathways to promote chondrocyte proliferation

**Fig. 3:**
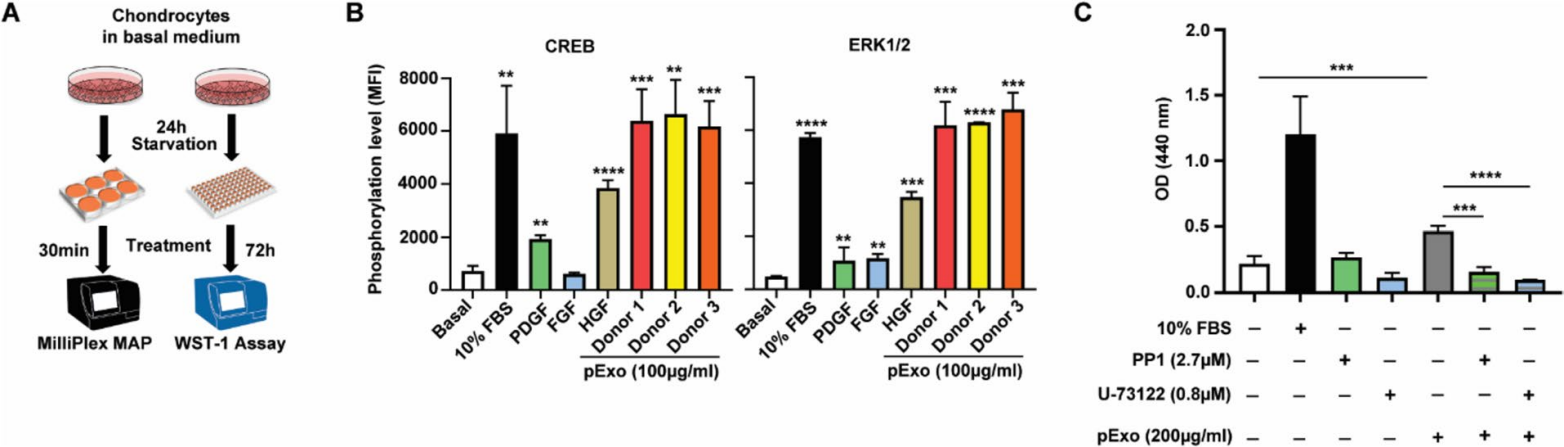
Impacts of pExo on signaling pathways. **A** In vitro study scheme. **B** Activation of CREB and ERK1/2 by 100 ng/ml PDGF, FGF, and HGF or 100 µg/ml pExo (*N* = 3 donors) was measured and quantified by their phosphorylation levels. Data is presented as mean ± 95% CI (*t*-test, vs basal medium condition, **P < 0.01, ***P < 0.005, ****P < 0.0001). **C** WST-1 assay of chondrocytes treated with 10% FBS, cell growth-related signaling pathway inhibitor PP1 or U-73122, or pExo (*N* = 3 donors) with indicated concentrations. Data is presented as mean ± 95% CI (*t*-test, ***P < 0.005, ****P < 0.0001)

Flow cytometry confirmed chondrocytes express PDGFR and HGFR, receptors of pExo-contained growth factors (Additional file 1: Fig. S4), suggesting pExo may display its effects via signaling pathways downstream of these factors. To interrogate this hypothesis, chondrocytes were treated and analyzed in cell signaling multiplex assays (Fig. 3A). Among analytes available for the assay, cAMP response element-binding protein (CREB) and extracellular signal-regulated kinase 1/2 (ERK1/2), which are reported to prevent chondrocyte apoptosis [37] and regulate chondrocyte differentiation [38], respectively, were significantly activated by pExo (Fig. 3B). We noticed 100 µg/ml pExo showed similar stimulatory effects on CREB and ERK1/2 activation compared to 10% FBS, which was stronger than any of the treated growth factors. Meanwhile, when co-treated with PP1 or U-73122, the pharmacological inhibitors of cell growth-related pathways, pExo’s pro-proliferative on chondrocytes, were abolished (Fig. 3C), suggesting partial mediation of these signaling pathways for pExo’s mitogenic effects in chondrocytes.

### pExo suppresses expressions of inflammatory and catabolic genes

**Fig. 4:**
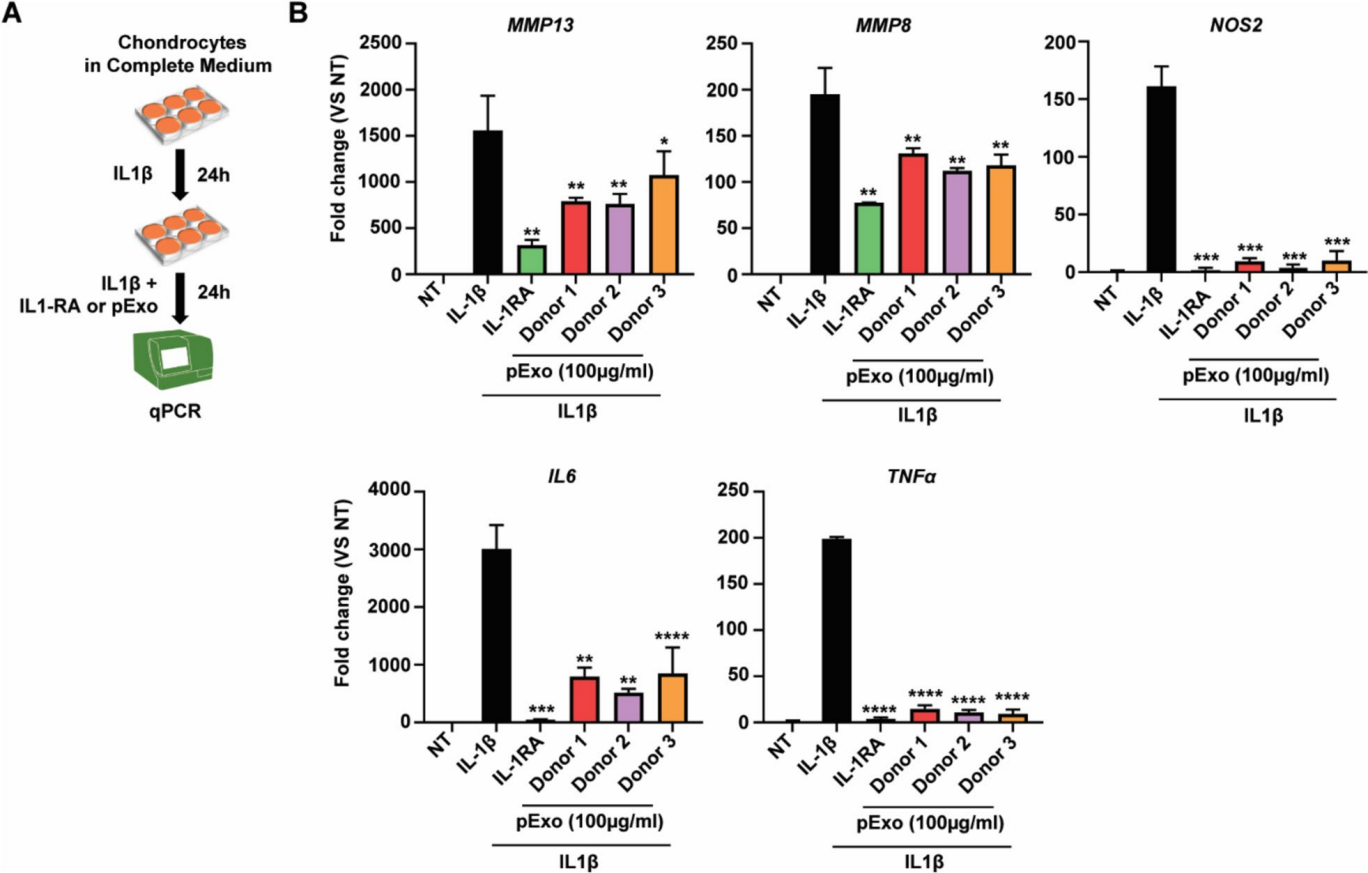
Gene expression of inflammatory and catabolic genes in chondrocytes. **A** In vitro model of IL1β stimulation: chondrocytes were treated with IL1β (1 ng/ml) for 24 h and then co-treated with pExo (100 µg/ml, *N* = 3 donors) or IL1-RA (1 µg/ml) for another 24 h before gene expression analysis by qPCR. **B** Expression levels of each gene were presented as mean ± 95% CI (vs IL1β treatment condition, *t*-test, *P < 0.05, **P < 0.01, ***P < 0.005, ****P < 0.0001)

To evaluate pExo’s ability of anti-inflammation and restoration of matrix synthesis, chondrocytes were treated by IL1β as a stimulus to mimic inflammatory environment of OA (Fig. 4A). Analysis of qPCR showed IL1β dramatically induced expression levels of catabolic genes matrix metallopeptidase (MMP)-13 and 8, inflammatory genes nitric oxide synthase 2 (NOS2), interleukin 6 (IL6), and tumor necrosis factor alpha (TNFα), which was reversed by its natural antagonist IL1-RA (Fig. 4B). Of note, pExo also significantly reduced the expressions of these genes stimulated by IL1β, suggesting its protective roles of reducing inflammation and matrix degradation in chondrocytes upon IL1β insult.

### *pExo alleviates OA-associated pain and restores cartilage damage *in vivo

**Fig. 5:**
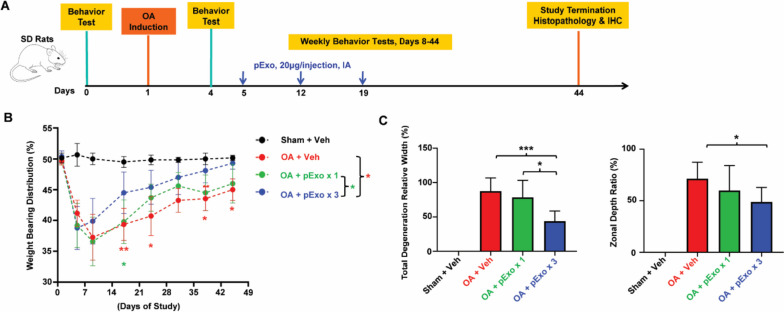
In vivo effects of pExo in a rat OA model. **A** Rat OA study scheme: animals received sham (Sham + Veh, *N* = 6 rats) or MCLT + MMT surgery were given with vehicle (OA + Veh, *N* = 11) or pExo by single (OA + pExo × 1, day 5, *N* = 10) or triple (OA + pExo × 3, days 5, 12, and 19, *N* = 10) IA injections. **B** Hind limb weight bearing capacity test was performed weekly, and data was presented as mean ± 95% CI (two-way ANOVA with Tukey’s multiple comparison tests, *P < 0.05 and **P < 0.01). **C** The severity of OA damage was histologically assessed by the relative width and depth of degenerated cartilage. Data was presented as mean ± 95% CI (one-way ANOVA with Tukey’s multiple comparison tests: Sham + Veh, *N* = 3 rats; OA + Veh, *N* = 8 rats; OA + pExo × 1, *N* = 7 rats; OA + pExo × 3, *N* = 7 rats; *P < 0.05 and ***P < 0.001)

Next, in vivo effects of pExo were evaluated in immunocompetent rats with surgery-induced OA (Fig. 5A). The results of static weight bearing tests indicated repeat doses of pExo significantly reduced OA-associated pain within 2 weeks post the first injection compared to vehicle and led to almost complete recovery by the end of study (Fig. 5B and Additional file 1: Table S3). However, while animals that received a single dose of pExo showed a trend towards recovery, it did not reach statistical significance. Histopathology analysis based on TB staining of the knee joints also demonstrated that cartilage degeneration was markedly reduced in the pExo repeat-dose group compared to the vehicle or single-dose group among OA rats with a trend of reduced osteoarthritic damage grade (Fig. 5C, Additional file 1: Figs. S5 and S6). In addition, the body weight growth of OA rats was not affected by single or repeat dosing of pExo (Additional file 1: Fig. S7). No unexpected clinical signs or deaths were observed throughout the study, while upon termination, no abnormalities or treatment-related changes were noted by gross examination (data not shown), indicating the potentially favorable tolerability and safety profile of pExo IA administration.

**Fig. 6:**
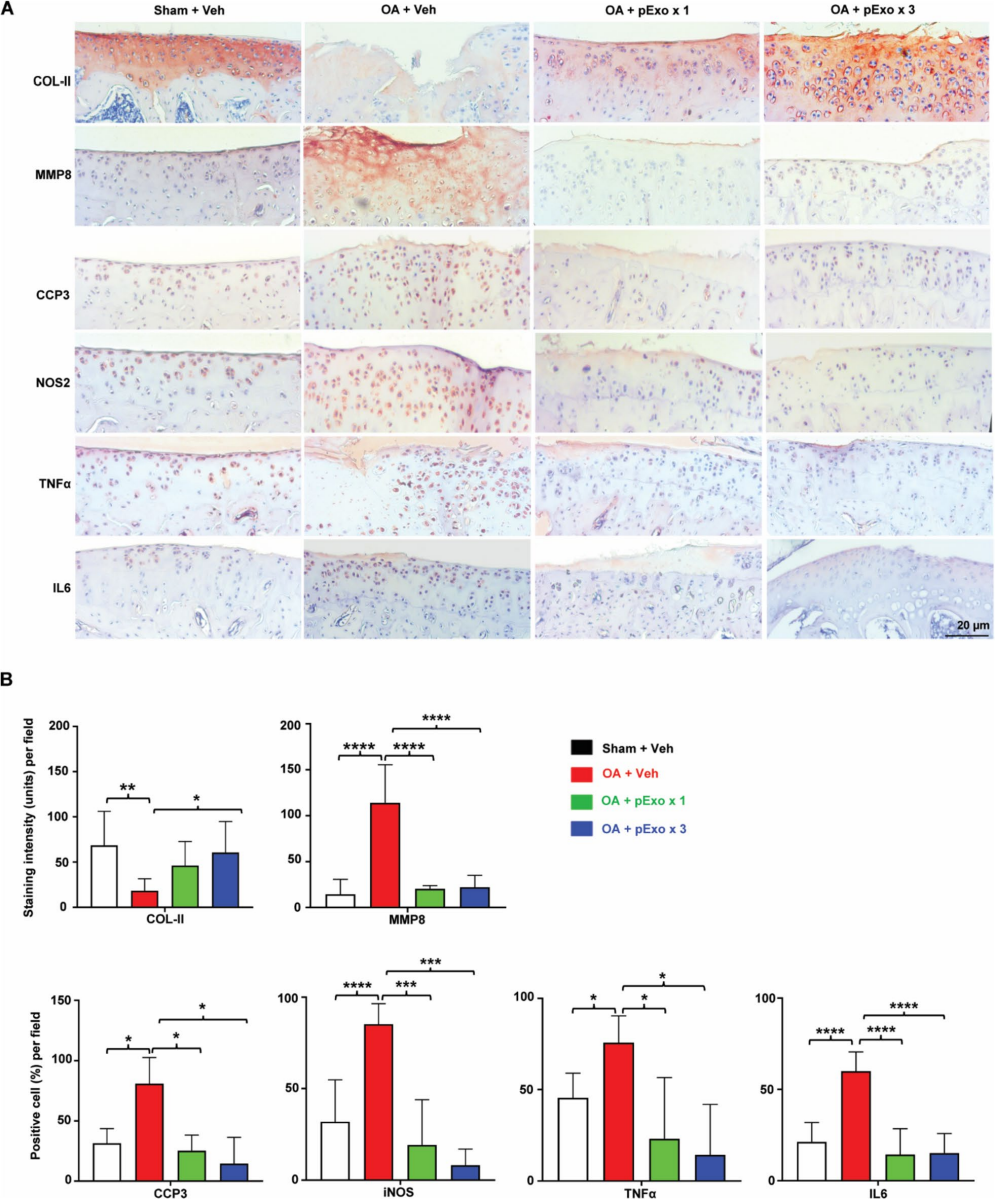
IHC of cartilage tissues. **A** Representative IHC images (*N* = 3 rats per group) of target proteins expressed in cartilage tissues. **B** IHC staining signals were quantified and presented as mean ± 95% CI (*N* = 3 rats per group, one-way ANOVA with Tukey’s multiple comparison tests, *P < 0.05, ***P < 0.001, and ****P < 0.0001)

The OA knee joint sections (Fig. 6A, B, Sham + Veh vs OA + Veh) upon IHC staining showed characteristic changes associated with the disease at protein level: significantly reduced expression of cartilage component collagen II (COL-II) as well as increased expressions of catabolic matrix metallopeptidase 8 (MMP8), cell death marker cleaved caspase 3 (CCP3), inflammatory mediator NOS2, and inflammatory cytokines TNFα and IL6. However, the dysregulation of these proteins was almost completely abolished by pExo treatment (Fig. 6B), which is consistent with the in vitro data and suggests pExo may alleviate OA pains and reduce cartilage loss by modulating expressions of these molecules. These data demonstrated the efficacy of pExo in the treatment of OA in a dose-dependent manner. It was also well tolerated with no adverse immune reactions from the use of xenogeneic human exosomes in immunocompetent animals being observed.

## Discussion

Our study demonstrates for the first time that pExo carries the defining characteristics of exosomes and a potential therapeutic candidate as a disease-modifying agent through modulating multiple targets of OA. In cultured chondrocytes, pExo promotes migration and proliferation in a dose-dependent manner via cell growth-related signaling pathways while simultaneously suppressing inflammation and catabolism. In animals, repeated doses of pExo alleviated OA pain, restored cartilage damage, and downregulated expressions of pro-inflammatory, catabolic, and apoptotic proteins. The mitogenic, pro-anabolic, anti-catabolic, and anti-inflammatory proteins contained in pExo may contribute to these effects.

In a rat model with surgically created osteochondral defects, repeat IA injections of 100 µg human embryonic MSC exosomes regenerated cartilage by enhancing cell proliferation and reducing apoptosis and inflammation [11, 39]. In another rat model of chemical-induced temporomandibular joint OA, repeat dosing of 100 µg MSC exosomes reduced pain, cartilage loss, and inflammation [40]. In our study using the MCLT + MMT rat OA model, 3 weekly IA injections of 20 µg pExo demonstrate similar therapeutic effects: pain alleviation, cartilage restoration, reduction of apoptosis, inflammation, and catabolism. Moreover, MSC exosomes increased chondrocyte migration and proliferation via the AKT and ERK signaling pathways as well as ameliorated IL1β-induced inflammatory effects and matrix degradation [39], which are also consistent with our in vitro data. Although pExo’s mechanism of action requires further investigation, the above consistent observations suggest the wide-ranging therapeutic efficacies of pExo against multiple OA targets overlap with MSC exosomes.

Developed as a novel therapeutic strategy, MSC exosomes are aimed to overcome clinical limitations [10] and operational challenges [11] of cellular therapies. For example, exosomes derived from Hoffa fat pad [41] or synovial fluid [42] MSCs showed promising chondroprotective effects both in vitro and in vivo. Unlike other human tissues, the placentas are of a consistent age, and would otherwise be discarded as medical waste. Manufactured from screened healthy donors with consistent yield and safety control, pExo generation does not require isolation, expansion, or maintenance of MSCs or any other cell types, thus greatly reducing cost and technical challenges. As an allogeneic approach, pExo can better address product consistency and provide timely availability for patients as an “off-the-shelf” therapeutic.

As a temporary organ that develops during pregnancy, the placenta not only consists of specific cell populations such as cytotrophoblast, syncytiotrophoblast, endothelial cells, and decidual cells but also has abundant hematopoietic and multipotent MSCs, which make placenta a unique source of MSCs with unique functional properties for therapeutic use compared to MSCs derived from other adult tissues [12–14]. Our earlier studies indicate MSC-like cells derived from human placental tissue possess immunomodulatory, anti-inflammatory, pro-regenerative, neuroprotective, and angiogenic properties in preclinical models of neuropathic pain, experimental allergic encephalomyelitis, and ischemic stroke [43–47]. Together with reported therapeutic efficacies of placental MSCs in preclinical and clinical OA studies [48], pExo’s beneficial properties may be at least partially attributed to the placental MSC population, which is one of pExo’s cellular resources.

Similar to exosomes secreted by cultured AFSCs [24], small extracellular vesicles obtained from chorionic-derived MSCs were also characterized to explore their potential in OA treatment [49]. Other biomaterials or biologics derived from human placentas demonstrated reduced pain, cartilage damage, and inflammation without product-related adverse findings in animal models of OA [18–23]. In patients, the efficacy and safety of placental tissue products have been revealed by recent clinical trials showing alleviation of OA symptoms such as joint pain and motion dysfunction without serious, unanticipated, product-related adverse events [50–53]. However, the lack of standardized process development and manufacturing criteria of these placental biomaterials or biologics may lead to product heterogeneity and inconsistent efficacies. With well-defined characteristics such as particle size and marker protein expression as an exosome product, pExo may address these issues in further studies.

The safety of exosomes derived from human MSCs or AFSCs in immunocompetent rats is suggested by the absence of observed treatment-related adverse effects [11, 24, 39, 40]. At 1-year follow-up, IA injection of extracellular vesicles isolated from allogeneic bone marrow-derived MSCs also appeared to be safe and clinically efficacious for OA patients [9]. Consistently, we also found multiple IA injections of pExo were well tolerated and safe in immunocompetent rats. Our unpublished data further demonstrates no immunogenicity or hepatotoxicity detected in an immunocompetent mouse model with repeat dosing of pExo by systematic infusion. However, as exosomes represent novel cell-free therapeutic agents in many conditions, there are still significant challenges and issues related to safety, manufacture, and regulation, which is highlighted by the fact that there are no FDA-approved exosome products to date [54]. Undoubtedly clinical studies are needed to further understand pExo’s therapeutic potential. Finally, although it is plausible to conclude pExo’s anti-OA effects are mediated by a myriad of components, future studies to better understand underlying mechanisms and further optimize pExo as a therapeutic agent in comparison with exosomes of other resources are warranted.

## Conclusions

Our study demonstrates for the first time that pExo carries exosome characteristics and displays multiple therapeutic effects in alleviating OA symptoms and modifying disease progression preclinically. Therefore, pExo can be further investigated and produced as a new anti-OA therapeutic under standardized process development and manufacturing criteria, with reduced cost and technical challenges compared to MSC exosomes.

### Supplementary Information

**Additional file 1: Table S1.** pExo size distribution. **Table S2.** Primary antibodies for immunohistochemistry. **Table S3.** Statistical analysis of rat OA study. **Fig. S1.** Full, uncropped gel and Western blot images of pExo (N=9 donors) to examine levels of CD63, CD81 and CD9. **Fig. S2.** WST-1 assay showed pExo of 3 donors promotes chondrocyte proliferation in a dose-dependent manner. Data is presented as mean ± 95% CI (t-test, vs Basal medium condition, **P<0.01, ***P<0.005, ****P<0.0001). **Fig. S3.** Representative images of Transwell migration using chondrocytes cultured in basal medium and treated with or without pExo for 24 hours. **Fig. S4.** Flow cytometry diagrams showing HGFR and PDGFR expressions in human chondrocytes. **Fig. S5.** Representative toluidine blue staining images of knee joint tissues collected from rat OA study. Note cartilage zones (C) were stained in blue purple and cartilage loss or degeneration was noted by yellow arrows. **Fig. S6.** Osteoarthritic damage scores were compared among treatment groups of OA rats. Data was presented as mean ± 95% CI and statistically analyzed by one-way ANOVA with Tukey’s multiple comparison tests. **Fig. S7.** Body weight change was calculated based on weekly measurements and presented as mean ± 95% CI. Although body weight growth rate of rats received MCLT + MMT OA surgery was reduced compared to sham rats overall, two-way ANOVA with Tukey’s multiple comparison tests was not able to detect any statistic difference in comparisons among three OA groups: OA + Veh, OA + pExo x 1 and OA + pExo x 3.

## Data Availability

All data needed to evaluate the conclusions in the paper are presented in the paper. Additional data related to this paper may be requested from the authors.

## Abbreviations

AFSCs: Amniotic fluid stem cells
CCP3: Cell death marker cleaved caspase 3
COL-II: Collagen II
CREB: CAMP response element-binding protein
ERK1/2: Extracellular signal-regulated kinase 1/2
FGF2: Fibroblast growth factor 2
HGF: Hepatocyte growth factor
IA: Intraarticular
IHC: Immunohistochemistry
IL-1RA: Interleukin-1 receptor antagonist
IL6: Interleukin 6
MCLT + MMT: Medial meniscal tear/medial collateral ligament tear
MFI: Median fluorescence intensity
MMP-8 and 13: Matrix metallopeptidase (MMP)-8 and 13
MSCs: Mesenchymal stromal/stem cells
NOS2: Nitric oxide synthase 2
NTA: Nanoparticle tracking analysis
OA: Osteoarthritis
OD: Optical density
PDGF: Platelet-derived growth factor
pExo: Placenta-derived exosomes
qRT-PCR: Quantitative real-time PCR
sICAM-1: Soluble intercellular adhesion molecule-1
TB: Toluidine blue
TIMP-1 and 2: Tissue inhibitor of metalloproteases-1 and 2
TNFα: Tumor necrosis factor alpha
