# Supplementary material for: Characterization of human placenta-derived exosome (pExo) as a potential osteoarthritis disease modifying therapeutic
Source: Arthritis Res Ther. 2023 Nov 28;25:229. doi: 10.1186/s13075-023-03219-z (PMC10683254; doi:10.1186/s13075-023-03219-z)
Supplement: Supplementary file 1 — Additional file 1: Table S1. pExo size distribution. Table S2. Primary antibodies for immunohistochemistry. Table S3. Statistical analysis of rat OA study. Fig. S1. Full, uncropped gel and Western blot images of pExo (N=9 donors) to examine levels of CD63, CD81 and CD9. Fig. S2. WST-1 assay showed pExo of 3 donors promotes chondrocyte proliferation in a dose-dependent manner. Data is presented as mean ± 95% CI (t-test, vs Basal medium condition, **P<0.01, ***P<0.005, ****P<0.0001). Fig. S3. Representative images of Transwell migration using chondrocytes cultured in basal medium and treated with or without pExo for 24 hours. Fig. S4. Flow cytometry diagrams showing HGFR and PDGFR expressions in human chondrocytes. Fig. S5. Representative toluidine blue staining images of knee joint tissues collected from rat OA study. Note cartilage zones (C) were stained in blue purple and cartilage loss or degeneration was noted by yellow arrows. Fig. S6. Osteoarthritic damage scores were compared among treatment groups of OA rats. Data was presented as mean ± 95% CI and statistically analyzed by one-way ANOVA with Tukey’s multiple comparison tests. Fig. S7. Body weight change was calculated based on weekly measurements and presented as mean ± 95% CI. Although body weight growth rate of rats received MCLT + MMT OA surgery was reduced compared to sham rats overall, two-way ANOVA with Tukey’s multiple comparison tests was not able to detect any statistic difference in comparisons among three OA groups: OA + Veh, OA + pExo x 1 and OA + pExo x 3. [file 13075_2023_3219_MOESM1_ESM.docx]

**Supplemental Materials**

**Characterization of human placenta-derived exosome (pExo) as a potential osteoarthritis disease modifying therapeutic**

Chenfei Huang^1^, Yuechao Zhao^1^*, Shengchen Lin^1^, Lin Li^1^, Xuan Guo^1^, Sebastian Yumiseba^1^, Jeng-dar Yang^1^, Robert Hariri^1^, Qian Ye^1^, Shuyang He^1^, Adrian Kilcoyne^1^

# **Supplemental Methods**

**Flow Cytometry**

Antibodies purchased from R&D systems were used to analyze expression of hepatocyte growth factor receptor (HGFR, Cat# FAB3582P) and Platelet-derived growth factor receptor (PDGFR, Cat# FAB1263P) in human chondrocytes. Analysis was performed using a BD FACS CANTO II flow cytometers, and the samples were analyzed using Diva software (BD Biosciences), while isotype controls were included.

## **Static Weight Bearing Test**

Static weight-bearing changes in rats were measured using an incapacitance tester at baseline (on Day 0), on Day 4 for stratification to obtain balanced groups, and on Days 8, 16, 23, 30, 37 and 44 before termination as the primary outcome measurements. Postural imbalance, which indicates a change in the pain threshold and weight distribution of the limbs, was determined. Each rat was placed so that each hind paw rests on a separate force plate on the incapacitance apparatus, and the weight borne by each hind limb was measured for 5 seconds. The mean of three consecutive measurements for each rat were recorded and data was calculated by the formula:

Weight bearing distribution = Operated hind limb / (Non-operated hind limb + Operated hind limb) %

In total, 37 of 38 rats were used for the study: one rat was removed since its test data on Day 4 was out of range.

## **Clinical Observations of Rats**

The animals were observed for toxic/adverse symptoms, during 1-week acclimation before MCLT + MMT surgery and throughout the study at least twice a week. Observations documented any irregularities, such as changes in local injection site, skin, fur, eyes, mucous membranes, respiratory, occurrence of secretions and excretions (e.g. diarrhea) and autonomic activity (e.g. decreased motor activity, salivation, piloerection, ptosis, unusual respiratory pattern). Changes in posture and response to handling, as the presence of bizarre behavior, tremors, convulsions, sleep and coma were also included. All observed abnormalities, toxic signs, moribund condition, and pre-terminal deaths were recorded.

## **Histopathology Analysis of Rat Knee Joints**

To evaluate histopathology, knee joint samples were fixed in 70% ethanol and then 4% formaldehyde before EDTA decalcification for up to 30 days. Then tissues were trimmed, put in embedding cassettes, and processed routinely for paraffin embedding. Three sections were cut from each knee and stained with toluidine blue to visualize cartilage distinction and histological evaluation of total cartilage degeneration relative width (% of cartilage degeneration area width divided by projected cartilage surface width) and zonal depth ratio of lesions (% degenerated cartilage depth divided by thickness of the projected cartilage surface to tidemark). In addition, the grade of osteoarthritic damage, a semi-quantitative scoring system recommended by OARSI, was also adapted for comparison.

**Supplemental Tables**

## **Supplemental Table 1. pExo size distribution**

| **pExo Donor** | **Mean diameter (nm)** | **Mode (nm)** |
| --- | --- | --- |
| 1 | 181 | 107 |
| 2 | 161 | 113 |
| 3 | 155 | 109 |
| 4 | 186 | 133 |
| 5 | 201 | 114 |
| Mean | 177 | 115 |
| SD | 49 | 10 |

**Supplemental Table 2. Primary antibodies for immunohistochemistry**

| **Antibody** | **Vendor** | **Catalog #** | **Dilution** |
| --- | --- | --- | --- |
| COL-II | Sigma-Aldrich | MAB8887 | 1:500 |
| MMP8 | Abcam | ab81286 | 1:200 |
| TNFα | Abcam | ab220210 | 1:200 |
| IL6 | Abcam | ab9324 | 1:200 |
| NOS2 | Abcam | ab283668 | 1:1000 |
| CCP3 | Cell Signaling Technology | 9661S | 1:200 |

**Supplemental Table 3. Statistical analysis of rat OA study**

| Comparison\Days | 0 | 4 | 8 | 16 | 23 | 30 | 37 | 44 |
| --- | --- | --- | --- | --- | --- | --- | --- | --- |
| Sham + Veh vs OA + Veh | 0.99 | ***0.0001*** | ***0.0001*** | ***0.0001*** | ***0.0001*** | ***0.0017*** | ***0.0022*** | ***0.022*** |
| Sham + Veh vs OA + pExo x 1 | 0.99 | ***0.0001*** | ***0.0001*** | ***0.0001*** | ***0.0049*** | 0.096 | ***0.015*** | 0.1 |
| Sham + Veh vs OA + pExo x 3 | 0.99 | ***0.0001*** | ***0.0001*** | ***0.033*** | 0.074 | 0.41 | 0.73 | 0.96 |
| OA + Veh vs OA + pExo x 1 | 0.99 | 0.57 | 0.97 | 0.99 | 0.22 | 0.43 | 0.93 | 0.92 |
| OA + Veh vs OA + pExo x 3 | 0.96 | 0.41 | 0.32 | ***0.0055*** | ***0.014*** | 0.077 | ***0.018*** | ***0.029*** |
| OA + pExo x 1 vs OA + pExo x 3 | 0.97 | 0.99 | 0.16 | ***0.017*** | 0.7 | 0.81 | 0.11 | 0.16 |

Two-way ANOVA with Tukey's multiple comparisons test was used to calculate P values of weight bearing test data collected from rat OA study as plotted in Figure 5B. Significant differences indicated according to P values are highlighted in bold italic font.

## **Supplemental Figure 1**

**
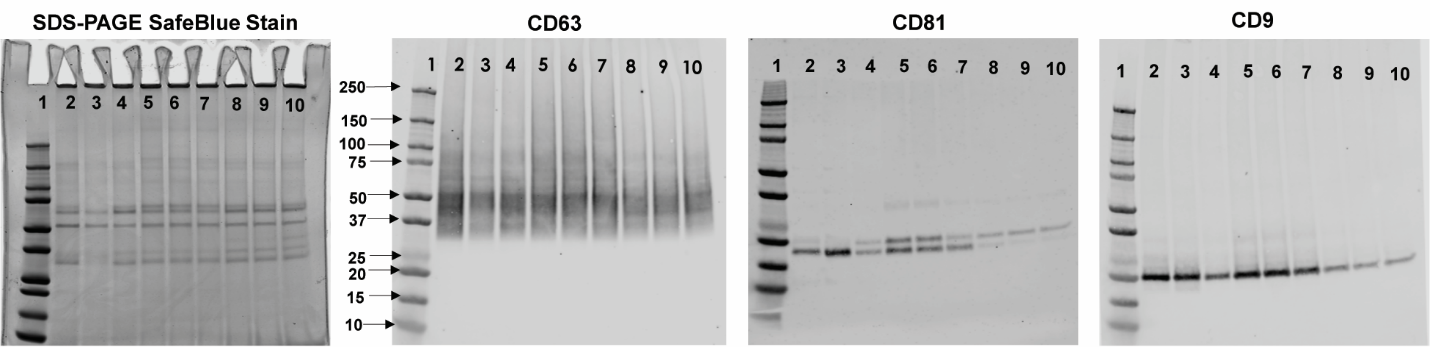
**

Full, uncropped gel and Western blot images of pExo (N=9 donors) to examine levels of CD63, CD81 and CD9.

## **Supplemental Figure 2**


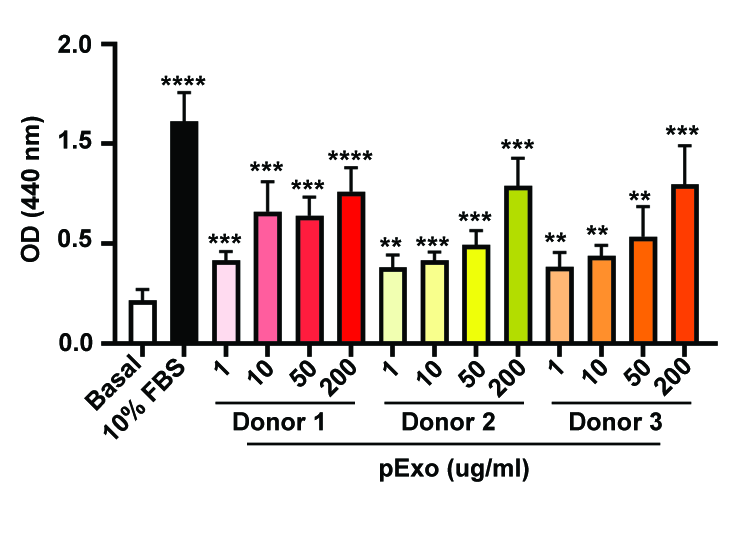


WST-1 assay showed pExo of 3 donors promotes chondrocyte proliferation in a dose-dependent manner. Data is presented as mean ± 95% CI (*t*-test, vs Basal medium condition, ***P*<0.01, ****P*<0.005, *****P*<0.0001).

## **Supplemental Figure 3**


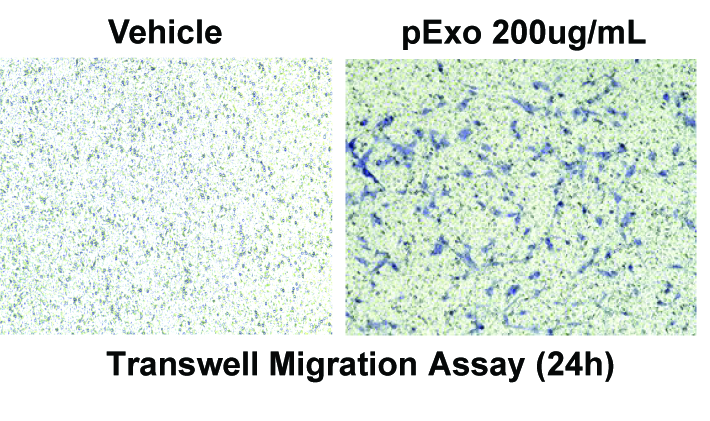


Representative images of transwell migration using chondrocytes cultured in basal medium and treated with or without pExo for 24 hours.

## **Supplemental Figure 4**


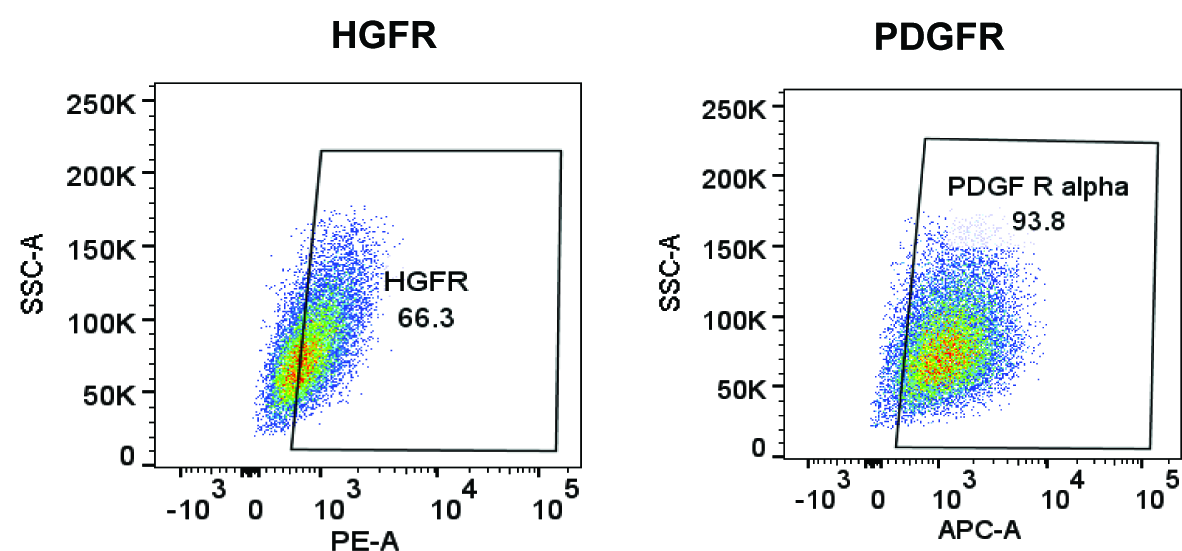


Flow cytometry diagrams showing HGFR and PDGFR expressions in human chondrocytes.

## **Supplemental Figure 5**


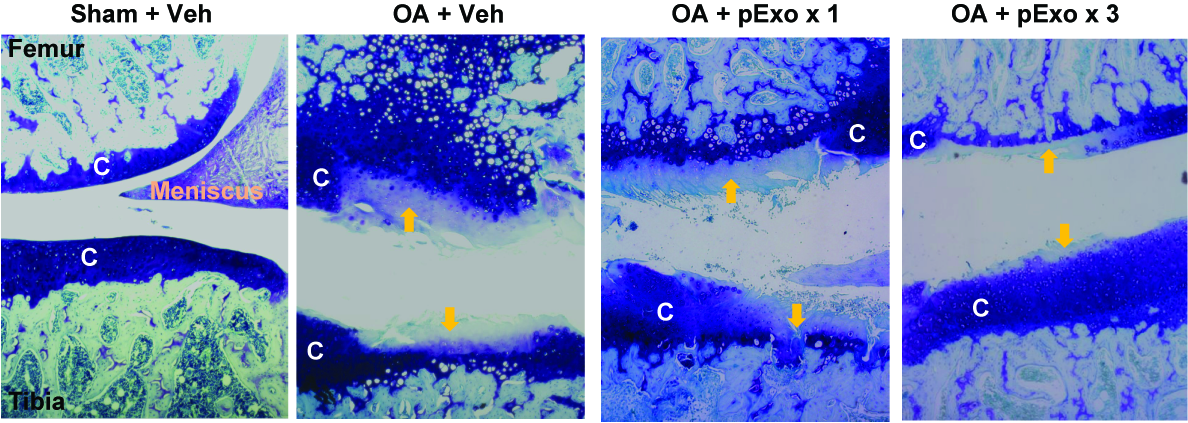


Representative toluidine blue staining images of knee joint tissues collected from rat OA study. Note cartilage zones (C) were stained in blue purple and cartilage loss or degeneration was noted by yellow arrows.

**Supplemental Figure 6**


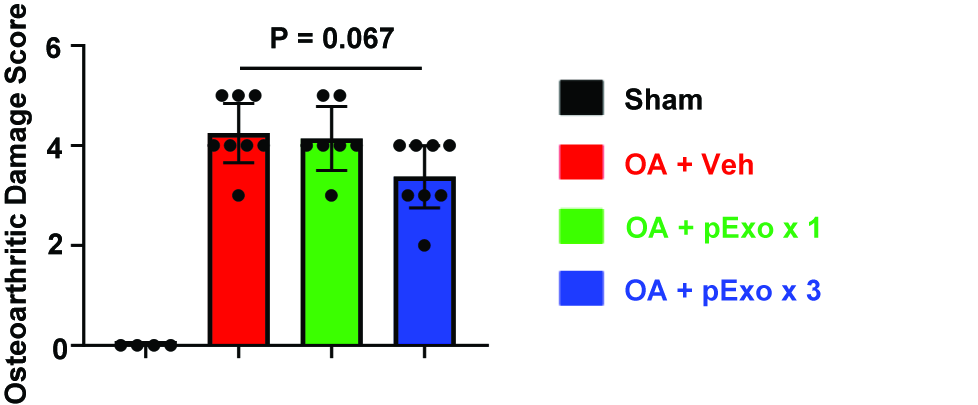


Osteoarthritic damage scores were compared among treatment groups of OA rats. Data was presented as mean ± 95% CI and statistically analyzed by one-way ANOVA with Tukey’s multiple comparison tests.

## **Supplemental Figure 7**


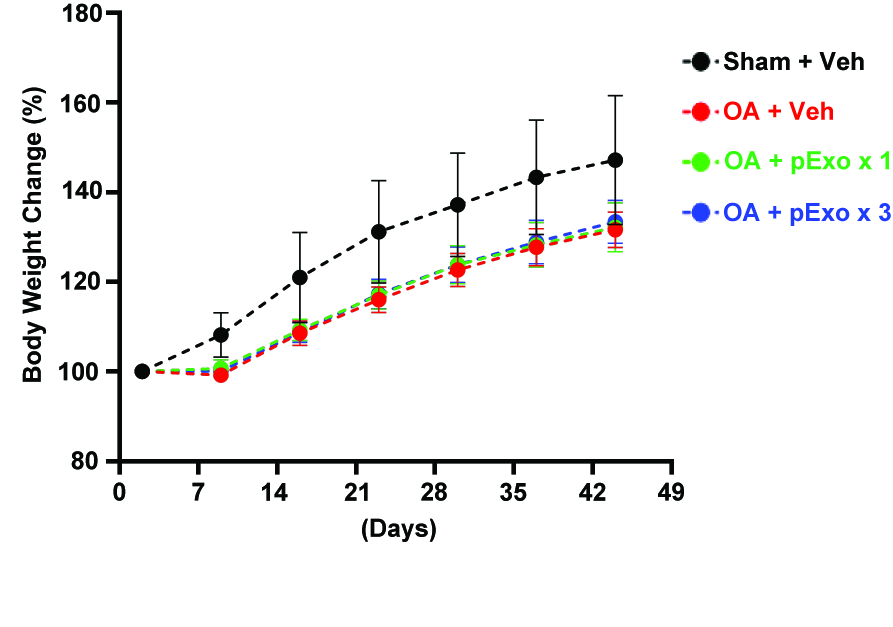


Body weight change was calculated based on weekly measurements and presented as mean ± 95% CI. Although body weight growth rate of rats received MCLT + MMT OA surgery was reduced compared to sham rats overall, two-way ANOVA with Tukey’s multiple comparison tests was not able to detect any statistic difference in comparisons among three OA groups: OA + Veh, OA + pExo x 1 and OA + pExo x 3.
